# Injection practice in Kaski district, Western Nepal: a community perspective

**DOI:** 10.1186/s12889-015-1775-5

**Published:** 2015-04-29

**Authors:** Sudesh Gyawali, Devendra Singh Rathore, Pathiyil Ravi Shankar, Vikash Kumar KC, Manisha Maskey, Nisha Jha

**Affiliations:** Department of Pharmacology, Manipal College of Medical Sciences, Pokhara, Nepal; Department of Pharmacy, L.R. Institute of Pharmacy, Jabli-kyar, Solan, India; Department of Pharmacology, Xavier University School of Medicine, Oranjestad, Aruba Kingdom of the Netherlands; Department of Statistics, PN Multiple Campus, Pokhara, Nepal; Department of Community Medicine, Manipal College of Medical Sciences, Pokhara, Nepal; Department of Clinical Pharmacology & Therapeutics, KIST Medical College, Imadol, Nepal

**Keywords:** Community, Injection practice, Knowledge, Needle-stick injury, Nepal

## Abstract

**Background:**

Previous studies have shown that unsafe injection practice is a major public health problem in Nepal but did not quantify the problem. The present community-based study was planned to: 1) quantify injection usage, 2) identify injection providers, 3) explore differences, if any, in injection usage and injection providers, and 4) study and compare people’s knowledge and perception about injections between the urban and rural areas of Kaski district.

**Methods:**

A descriptive, cross-sectional mixed-methods study was conducted from July to November 2012, using a questionnaire based survey and focus group discussions (FGDs). A semi-structured questionnaire advocated by the World Health Organization was modified and administered to household heads and injection receivers in selected households and the FGDs were conducted using a topic guide. The district was divided into urban and rural areas and 300 households from each area were selected. Twenty FGDs were held.

**Results:**

In 218 households (36.33%) [99 in urban and 119 in rural] one or more members received at least one injection. During the three month recall period, 258 subjects (10.44%) reported receiving injection(s) with a median of two injections. The average number of injections per person per year was calculated to be 2.37. Health care workers (34.8%), staff of medical dispensaries (37.7%), physicians (25.2%), and traditional healers (2.3%) were consulted by the respondents for their basic health care needs and for injections. Compared to urban respondents, more rural respondents preferred injections for fever (p < 0.001). People preferred injections due to injections being perceived by them as being powerful, fast-acting, and longer lasting than oral pills. More than 82% of respondents were aware of, and named, at least one disease transmitted by using unsterile syringes during injection administration or when syringes are shared between people.

**Conclusions:**

Less preference for injections and high awareness about the association between injections and injection-borne infections among the general population is encouraging for safe injection practice. However, respondents were not aware of the importance of having qualified injection providers for safe injections and were receiving injections from unqualified personnel.

**Electronic supplementary material:**

The online version of this article (doi:10.1186/s12889-015-1775-5) contains supplementary material, which is available to authorized users.

## Background

Injection is one of the many important health care procedures used worldwide in the allopathic system of medicine. It is perceived by both prescribers and patients as being one of the most powerful methods of restoring or maintaining health, making it popular and sometimes overused in developing countries [1-3]. The unsafe use of injections, particularly reuse of injection devices without proper sterilization, was high (almost 75%) in South East Asia Region D which includes Nepal [2].

Various factors influence prescription and use of injections. The attitude of patients towards injections [4,5], the qualification of injection providers, and the availability of quality injections (with or without prescriptions) and other equipment, have significant influence on injection use (popularity) [1,6]. Injection providers perceive that injections are preferred by patients; its use elevates their status and helps them to prove their professional credibility and compete in a competitive health market [7-9]. Various types of health care personnel with different qualifications and training administer injections. These personnel differ from place to place and time to time [10]. Studies [2,11] have reported that, in developing countries including Nepal, significantly large numbers of injections are given by untrained and unqualified health workers or employees of government health care facilities [1]. In Nepal there are no specific rules regarding the qualification and training of injection providers [12].

### Safe and unsafe injection practice

A safe injection is one that, “does not harm the recipient (patient), does not expose the health care worker to any risk and does not result in waste that is dangerous for the community” [13]. Hence, the safety of an injection should be evaluated with respect to these three aspects, that is, safety of recipient, the injection provider, and the community. The recipients of injections could be safeguarded by administering useful injections only (rational prescription) with a new sterile single use device and observing proper techniques by a qualified well-trained health care worker [14,15].

The use of unnecessary and unsafe injections contributes to the transmission of a wide variety of blood-borne pathogens, including viruses, bacteria, fungi and parasites [16]. Among the infections transmitted through such practice, Human immunodeficiency virus (HIV), Hepatitis B virus (HBV) and Hepatitis C virus (HCV) are of special concern as the social, economic, and disease burden related to these infections are high, the infections are preventable, and the treatment is very costly and often not available to the people in need [17]. Unfortunately these problems are overlooked in developing countries because the immediate (acute) effects of unsafe injections are not common and often go unnoticed [13,18].

### Health care services in Nepal

Nepal is a developing country situated in the South East Asia region, lying between two giants (India and China). Health care services in Nepal are provided by both the government and the private sector. Government delivers its services through the organizational network of the Department of Health Services (DoHS). A Sub Health Post (SHP) is the first contact point for basic health care services. Each level above the SHP is a referral point in the network: from SHP to Health Posts (HP) to Primary Health Care Centers (PHCC) to district, zonal & regional hospitals and finally to tertiary care hospitals in the capital of Nepal (Kathmandu). These units are managed by trained health care workers [e.g. Auxiliary health workers (AHW), Auxiliary Nursing midwifes (ANM), Community Health Workers (CHW)], with medical doctors and nursing staff stationed at PHCC and other higher level hospitals [19]. The AHW have either health assistant (HA) or community medical auxiliary (CMA) qualification. The HAs and CMAs undergo basic medical training for 36 months and 18 months, respectively, after completing 10 years of schooling. They are trained to diagnose and treat common illnesses and refer patients for more specialized care if required [20,21]. ANMs obtain basic nursing training of 18 months after 10 years of schooling and assist in delivery of babies.

There are no compulsory injections that all citizens and/or residents in Nepal have to take. Government of Nepal provides free immunization services through routine and supplemental immunization programs [19]. The government encourages the community to ensure that all children up to the age of five are fully immunized [19] but it has not been made mandatory to have these vaccinations. Additionally, medical services provided by private hospitals (nursing homes), private teaching hospitals, private clinics, and missionary hospitals complement the government services [19].

Kaski district is one of the more advanced districts and favorite tourist destinations of Nepal [22]. All types of injections e.g. disposable syringe, cannula, prefilled syringes, insulin syringe, insulin pen etc. are available in the district. Except for a few narcotic injections, most injections and injection equipments (e.g. syringe, cannula etc.) can be purchased even without a prescription [21]. Insulin pen is also available in the district but is expensive. The type of injections available in a pharmacy, and health facility depends on the nature of the facility and its location.

A few studies related to injection practice in Nepal [12,23,24] have shown that unsafe injection practice and improper sharps management were a major public health problem. These studies were conducted in health care facilities, among health care workers (HCWs) and/or patients visiting the health facilities and did not quantify the problem at the population (community) level. Furthermore, a small scale study [25] had recommended a community-based survey to triangulate the initiatives taken by the government of Nepal and obtain a clearer picture of injection practices. The present community-based study was planned to: 1) quantify injection usage, 2) identify injection providers, 3) explore differences, if any, in injection usage and injection providers among urban and rural areas, and 4) study and compare people’s knowledge and perception about injections between urban and rural areas of Kaski district.

## Methods

### Study design

A descriptive cross sectional study employing both quantitative and qualitative research methods was conducted from July to November 2012. The study consisted of two components: a questionnaire based survey and focus group discussions (FGDs) with the respondents.

The questionnaire was administered to household heads and injection receiver/s (as explained later in the methodology section) in each household. Respondents who were willing to share their experiences and perceptions about diseases, therapy, and injection were invited to participate in FGDs. The FGD was planned on the same day or on the next day after the households’ survey in a particular enumeration area (EA). A ward, the smallest political division of the district with clear geographical boundaries, was considered as an EA.

### Study area

The research work was carried out in Kaski district of Nepal. For administrative purposes, the country is divided into 75 districts, 3915 Village Development Committees (VDCs) and 58 municipalities [26]. Kaski district is one of the 75 districts located in the Western region which has 43 VDCs and 2 municipalities (Pokhara and Lekhnath) [22]. The preliminary census report 2011 showed that the total population of the district was 490,429 [27].

### Sample size and sampling technique

The sample size was calculated using the formula [28]: Minimum sample size required (n) = 4pq/E^2^. Where ‘p’ (proportion of injection use), was calculated using the data from the pilot study carried out in Baglung (adjacent to Kaski) district. The calculated p value was 37% (0.37), q (1-p) was 0.63, and E (allowable error at 11%) was 0.0407. Hence, the required number of minimum households was calculated to be 563. Therefore, 600 households (300 from each stratum) were considered for the study.

The sampling was done using stratified sampling method dividing the district into two strata e.g. urban and rural. The municipalities of the district were classified as urban while the VDCs were stratified as rural. The total EAs (wards) in Kaski district were 420 and to cover more than 10% of total EA, it was decided to take 50 EAs (25 from urban and 25 from rural) for the study. Twelve households from each EA made a total of 300 households (25 EA × 12 households = 300 households).

All wards of VDCs were tabulated in the sequence as recommended by the district statistics office and mentioned in the district profile, Kaski [22]. The total number of households in each ward was written in adjacent columns of the table with cumulative total in the next column. The sample interval was calculated by dividing the total cumulative household in each stratum by 25 and a random number was generated using Excel 2013. The first three digits after the decimal were considered to be a random number. The ward corresponding to the household of random number generated and the numbers obtained after every addition of sample interval were selected for the study. In the sampled ward (EA), one house was selected randomly then consecutive households were covered until 12 households were covered.

For the purposes of this study, an injection was defined as *“a skin-piercing event performed with a syringe and/or needle with the purpose of introducing a curative substance or a vaccine into a patient by various routes*” [29]. The study included all type of injections e.g. therapeutic bolus injections and infusions, immunizations etc. Other skin-piercing events done using a needle e.g. to transfuse blood, phlebotomy for diagnostic purposes, injections for drug abuse etc. were excluded from the study.

#### Inclusion and exclusion criteria

Each household of Kaski district was considered a study unit. People who shared the same kitchen for at least six months were considered a family (household) [30]. Only HH who may or may not have received injection/s and the members of the family (household) who had received at least one injection during the last three months were considered for the survey. During the study period, there was a national Measles Rubella (MR) vaccination campaign in Kaski district. The MR vaccine administered during the national vaccination campaign was excluded from the study. People staying in Kaski for less than 6 months and children of age less than 6 months were also excluded from the study. A non-resident Nepalese family member who recently visited his/her family at Kaski and was sharing kitchen for less than six months was also not considered as a family member and was excluded [30].

### Data collection tools

Three data collection tools were used in the study: a basic household information form, a semi-structured questionnaire and a topic guide for FGDs (described in the FGD subsection). These are described below.

#### The basic household information form

This was used to collect basic information from the household head, e.g. age, gender, education, occupation, injection event during the last three months etc. of all the members of the household.

#### The questionnaire

The questionnaire used was based on the questionnaire advocated by the WHO to assess and evaluate injection practices [31]. The WHO advocated questionnaire was standardized as per the context. The questions were discussed among the authors and experts in the field, some questions were deleted, a few were modified, some were reframed and restructured for better understanding, and a few questions were added. Questions intending to collect information about exposure to mass media, their use to obtain health related information, and about health facilities preferred by the community for basic health care problems and injections were added. The questionnaire thus constructed was modified by including suggestions obtained during the pilot study and inputs from experts in the subject. The modified questionnaire was forward translated into Nepali (national) language by a group of three individuals proficient in both languages. The questionnaire translated into Nepali was then provided to a different group of three individuals proficient in both languages for backward translation to English. The backward translated English questionnaire was then compared with the original questionnaire and discrepancies, if any, were noted and analyzed. If required modifications to the original questionnaire were carried out and the same was finalized. The persons involved in the translation were not involved in the study.

The finalized questionnaire consisted of a mixture of structured close-ended and semi-structured open-ended questions and could be divided into 5 parts: part one collected information about the number of injections received (both therapeutic and vaccine) in the last three months; part two collected information about injection providers, types of syringe used for the injections, where the injection event took place and the money paid by the recipient for the injection; part three collected information about needle stick injuries (NSI); part four assessed the respondents’ knowledge and perception about safe injection practice, their preference of dosage form for fever and of health facilities for minor illness and injections; and part five collected information about exposure to mass media like radio, television, internet and newspapers, and use of these as sources of health information.

The pilot study was conducted during May and June 2012 in 60 households (10% of total sample size). The results of the pilot study were not included in the main analysis.

### Descriptions and measurement of some variables mentioned in the study

#### Age

Age was measured as years completed by a person in case of an adult or months completed in case of infants (age less than a year) at the time of the survey. The age was classified into five groups: Infants (up to 1 year), Toddlers (1–5 years), Children (5–14 years), adult (15–60 years) and elderly (more than 60 years).

#### Education

Central Bureau of Statistics (CBS) – Nepal states that a person is literate if s/he is able to read and write with understanding in any language and to perform simple arithmetic calculations [30]. In the study, people were classified into two groups: Illiterate and Literate.

People who had learned to read and write through literacy classes or at home were considered to be literate. People who had gone to school were considered educated. In terms of level of education, the respondents were classified into five groups: primary level completed (up to five years of education), secondary level completed (ten years or less), Higher secondary (up to 12 years of education), Graduation and Post-graduation and above.

#### Prevalence of injection use at the household level

The prevalence of injection use at the household level was calculated by as per the criteria mentioned by Staa et al. [6]:

Prevalence = Number of households in which at least one family member had received at least one injection in the past three months/Total number of the households considered for the study.

#### Frequency of injection use

Frequency of injection use was expressed as number of injections per person per annum which was calculated using the following formula [31]:

$$ \mathrm{Number}\ \mathrm{of}\ \mathrm{in}\mathrm{jections}\ \mathrm{per}\ \mathrm{per}\mathrm{son}\ \mathrm{per}\ \mathrm{annum}=\frac{\mathrm{Total}\ \mathrm{number}\ \mathrm{of}\ \mathrm{in}\mathrm{jections}\ \mathrm{received}\ \mathrm{in}\ \mathrm{the}\ \mathrm{last}\ \mathrm{three}\ \mathrm{months}}{\mathrm{Total}\ \mathrm{number}\ \mathrm{of}\ \mathrm{participants}\ \mathrm{of}\ \mathrm{the}\ \mathrm{survey}} \times 4 $$

#### Mass media exposure

Mass media exposure is a composite measure. It has been shown that people who have been exposed to mass media are more likely to know about HIV/AIDS, Sexually transmitted diseases and other health related issues than those who are not exposed [32]. It was computed based on whether the respondent listens to radio daily, watches television daily, reads newspapers at least once in a week and surfs internet at least once in a week. Exposure to media was divided into four categories: exposed to any one, exposed to any two, exposed to any three and all four.

### Data collection procedure

#### The survey

In each household, the head was asked the age, gender, education, religion and occupation of all the family members which was entered into the basic household information form. The head and all the members (present at the time of survey) were also asked whether they had received any injection(s) during the last three months. The person in a family (house) who makes health related decisions of the family was considered as household head (HH) for the study purposes. In the absence of the head, second active member of the family was consulted for the same.

The HH and the family members who had received at least one injection during the past three months were surveyed using the questionnaire in Nepali language. When the injection receiver was less than 14 years or could not communicate properly due to any disability, the questions were asked to the parent (preferably mother). In this group of injection receivers the questions from part one and two were only asked. The survey was preferably conducted on Saturday (official weekly holiday in Nepal) or in the evening, so that a maximum number of family members were present.

#### The FGDs

##### Conceptualization

The topics to be addressed during the FGD were conceptualized following a thorough literature review and discussion among the authors of this study and with inputs from experts in this field.

##### Creating the FGD guide

The topic guide was prepared by conducting a thorough literature review and explored certain issues which were noted during the survey in more detail. The guide covered a range of issues: population perspectives about illnesses and its treatment; role of injections in therapy; population preferences for injections; and knowledge about safe/unsafe injection practice.

##### Logistics and facilitation

A final year student of Bachelor of Pharmacy course was trained as a note taker. The note taker was responsible to take down hand written notes and observations (nonverbal behavior) during the FGDs. He was also responsible for audio recording when the discussions were recorded. The discussions were facilitated by SG (corresponding author of the manuscript) with experience of qualitative research and were held under the guidance of VKKC (coauthor).

Members eager to share their opinion and able to express their view fluently (in Nepali) were selected and requested to participate in FGDs. The discussions were conducted as per the convenience of the participants preferably during early or late afternoon. FGDs were conducted to get additional information regarding certain responses in the questionnaire (reasons for their practice/response) and covered the range of issues mentioned previously. Twenty (10 from urban and 10 from rural area) FGDs were conducted in different EAs. FGDs were held with 6–8 household members (age more than 15 years) from different households. A total of 147 participants participated in the twenty FGDs. The discussions were held for about 60–90 minutes without any break. The FGDs were conducted in Nepali language. Most FGDs were audio recorded while hand-written notes were also taken during some depending on the choice and consent of the participants. Immediately after the FGD, the facilitator along with the note taker checked and discussed the completeness of the notes.

##### Analysis and reporting

The qualitative data obtained from FGDs were coded and analyzed using deductive content analysis [33,34] where literature review and the FGD guides were used to develop categories and subcategories for coding and analysis. Audio recordings were transcribed in Nepali. The transcript was then translated into English by a person fluent in both languages and not associated with the study. Each transcript was read through several times to obtain a sense of the whole. All words, sentences and paragraphs containing aspects related to each other through their content were then coded for correspondence with predetermined categories. Additional information provided by the respondent was placed in a different new category if required. Direct quotes were contextualized, rendered readable and presented in the habitual language of the respondents. Finally, the quotes were translated from Nepali to English.

### Ethical issues

The study protocol was approved by the ethical review board of Nepal Health Research Council, Kathmandu, Nepal. The participants were explained about the study and were assured about the confidentiality of information given by them. Participants were given a clear option not to take part if they wished to do so. Informed verbal consent was obtained prior to the study. The consent for study subjects who were less than 14 years of age or could not communicate properly due to disability was taken from the accompanying parent (preferably the mother).

### Data analysis

All quantitative data was coded and entered into computer software programs Statistical Package for Social Sciences (SPSS) version 17.0 for Windows and Microsoft Excel 13 for statistical analysis. The data was presented as mean, median, inter-quartile range and standard deviation (SD) for continuous variables such as age, number of injections received etc. Frequency and percentage (%) of various variables were calculated for categorical variables like gender and incidence of NSI. Data from urban and rural areas were compared and Pearson chi-square test was done to check for significant relations. A p-value of <0.05 was considered as statistically significant.

## Results

The survey covered 2470 people (family members) in 600 households sampled. The mean ± SD family members in a household were 4.12 ± 1.75. The overall male: female ratio was 0.91, while the ratio in urban and rural areas was 0.89 and 0.94, respectively.

In the urban areas surveyed, 1196 family members were present in 300 households whereas in rural areas 1274 members were present in 300 households. Table 1 shows the comparative demographic characteristics of the urban and rural population in our survey.

**Table 1:** **Comparison of demographic characteristics of the surveyed respondents**

**Characteristics**		**Urban population n = 1196 (%)**	**Rural population n = 1274 (%)**	**Total population n = 2470 (%)**	*Chi-square (p) value
Gender	Female	632 (52.84)	658 (51.65)	1290 (52.23)	0.353 (>0.05)
	Male	564 (47.14)	616 (48.35)	1180 (47.77)	
Age in years	<1	3 (0.25)	10 (0.78)	13 (0.53)	20.935 (<0.001)
	1 to 4	50 (4.18)	61 (4.79)	111 (4.49)	
	5 to14	214 (17.89)	222 (17.43)	436 (17.65)	
	15 to 60	849 (70.99)	837 (65.70)	1686 (68.26)	
	>60	80 (6.69)	144 (11.30)	224 (9.07)	
Literacy	Illiterate	86 (7.19)	179 (14.05)	265 (10.73)	41.922 (<0.001)
	Literate	1090 (91.14)	1048 (82.26)	2138 (86.56)	
	Not applicable**	20 (1.67)	47 (3.69)	67 (2.71)	
Education	Primary	186 (15.55)	257 (20.17)	443 (23.46)	114.750 (<0.001)
	Secondary	353 (29.52)	405 (31.79)	758 (40.15)	
	Higher secondary	170 (14.21)	109 (8.56)	279 (14.78)	
	Graduation	218 (18.23)	91 (7.14)	309 (16.37)	
	Post-graduation	82 (6.86)	17 (1.33)	99 (5.24)	
Occupation	Farming	68 (6.00)	305 (25.50)	373 (16.01)	271.983 (<0.001)
	Student	470 (41.45)	462 (38.63)	932 (40.00)	
	Housewife/ unemployed	190 (16.75)	235 (19.65)	425 (18.24)	
	Business	166 (14.64)	57 (4.77)	223 (9.57)	
	Private employee	124 (10.93)	48 (4.01)	172 (7.38)	
	Government employee	81 (7.14)	29 (2.42)	110 (4.72)	
	Others	35 (3.09)	60 (5.02)	95 (4.08)	

*Chi-square & p values (two-sided) were calculated by Pearson chi-square test, **Children of age less than or equal to three years are not sent to school so literacy was not applicable to them.

### Injection use

Out of 600 households, in 218 (36.33%) [99 (33%) in urban and 119 (39.67%) in rural] of the households one or more members received at least one injection during the preceding three months. During the period, 258 (10.44%) of the subjects in the survey reported having received injection(s). One hundred and thirteen persons (9.45%, n = 1196) from urban areas and 145 persons (11.38%, n = 1274) from rural areas received at least one injection. The most frequently used single dose injection was Tetanus toxoid (TT) injection followed by long-acting (three months) contraceptive injection. Median number of injections received by the respondents was 2 with an inter quartile range of 2. The median (IQR) number of injections received by respondents in urban and rural areas was 1 (2) and 2 (2), respectively. The total number of injections used in the last three months by the sampled population was 1462 injections. Hence, the average number of injections per person per year was calculated to be 2.37.

Out of 1462 injections, only 42 (2.87%) were vaccines. The mean (± SD) age of injection receivers was 32.99 (±21.55) years. Table 2 shows injections were most commonly used in children age less than 5 years and elderly people of age more than 60 years.

**Table 2:** **Percentage of injection receivers classified according to age and place of residence**

**S. No.**	**Age in years**	**Percentage of population receiving injection**		
		**Urban area (n)**	**Rural area (n)**	**Total (n)**
**1.**	**Less than 1**	66.67 (3)	100.00 (10)	92.30 (13)
**2.**	**1 to 4**	16.00 (50)	11.47 (61)	13.51 (111)
**3.**	**5 to 14**	5.14 (214)	3.60 (222)	4.36 (436)
**4.**	**15 to 60**	9.42 (849)	12.19 (837)	10.79 (1686)
**5.**	**More than 60**	15.00 (80)	12.5 (144)	13.39 (224)

### Obtaining medical help

All HHs were asked, “Where and whom do you consult for your (families’) basic health care needs?” Health care workers (HCWs) at government health care facilities or at their clinics (34.8%), staff of medical dispensaries (37.7%), physician (25.2%), and traditional healers (2.3%) were consulted by the respondents for their basic health care needs and for injections. More than half (57.7%) of the respondents from rural areas stated that they preferred to consult HCWs, followed by staff of medical dispensaries (22.3%), for their health care needs and injection while 53% of urban respondents preferred staff of medical dispensaries followed by physicians (34.7%). Significantly (p < 0.001), higher number of rural respondents (4.3%) also preferred to consult traditional healers for basic health care need and for injections.

### Demographic characteristics of the people included in the detailed study of injection practice

To study the details of injection practice (including knowledge and perception of people towards injection practice), household heads and injection receivers aged 15 years and above were included. A total of 714 (354 from urban and 360 from rural) respondents satisfied the inclusion criteria and were included in the study. The mean (±SD) age of the respondents was 43.78 (±15.80) years with a range of 15 to 89 years.

In each household, information about all the family members was collected using the basic household information form (Refer to Additional file 1). In 600 households, a total of 2470 family members were present; information about the household members is presented in Table 1. Out of the 2470, 258 received injections during the last three months recall period which included children and adults (>15 years of age). The questionnaire was administered to all 258 injection receivers. Injection receivers less than 14 years of age were administered part1 and 2 of the questionnaire and the information was collected from the parent (preferably the mother).

The questionnaire was also administered to household heads (HHs) who may or may not have received injection during the last three months. The household heads who received injection were requested to answer all questions while among the heads who did not receive injections part 1 of the questionnaire was excluded. The demographic information relating to household heads and injection receivers aged more than 15 years are presented in Table 3. The heads and injection receivers (>15 years of age) who were willing to share their experience and opinion were selected for FGDs.

**Table 3:** **Percentage of respondents classified by place of residence according to selected demographic characteristics**

**Characteristics**		**Urban, frequency (%), n = 354**	**Rural, frequency (%), n = 360**	**Total (%), n = 714**	*Chi-square (p) value
Gender	Male	213 (60.17)	206 (57.22)	419 (58.68)	0.639 (0.424)
	Female	141 (39.84)	154 (42.78)	295 (41.32)	
No. of respondents who received injections in last 3 months**.		92 (25.99)	119 (33.06)	211 (29.55)	4.282 (0.039)
Literacy	Literate	324 (91.53)	294 (81.67)	618 (86.55)	14.907 (<0.001)
	Illiterate	30 (8.47)	66 (18.33)	96 (13.45)	
Education^#^	Primary	7 (2.43)	50 (23.36)	57 (11.35)	92.714 (<0.001)
	Secondary	108 (37.50)	110 (51.40)	218 (43.43)	
	Higher secondary	62 (21.53)	28 (13.08)	90 (17.93)	
	Graduation	66 (22.91)	22 (10.28)	88 (17.53)	
	Post-graduation	45 (15.63)	4 (1.87)	49 (9.76)	
Occupation	Farming	28 (8.14)	166 (47.16)	194 (27.87)	177.276 (<0.001)
	Student	26 (7.56)	12 (3.41)	38 (5.46)	
	Housewife/unemployed	65 (18.89)	70 (19.89)	135 (19.40)	
	Business	96 (27.91)	41 (11.65)	137 (19.68)	
	Private employee	54 (15.70)	13 (3.69)	67 (9.63)	
	Government employee	52 (15.12)	13 (3.69)	65 (9.34)	
	Others	23 (6.68)	37 (10.51)	60 (8.62)	

*Chi-square and p value (two-sided) were calculated by Pearson chi-square test comparing urban and rural frequencies; **The injection receivers were of age ≥ 15 years; ^#^ *The percentage was adjusted as per the number of literate in the respective groups.*

Out of 714 respondents, 618 (86.55%) were literate and the difference in literacy among urban and the rural population was highly significant (p < 0.001). Out of 618 literate people, 502 (81.22%) had formal education. The number of people having formal education and level of the education achieved were also significantly different (p < 0.001) in urban and rural areas (Table 3).

#### Injection providers

All respondents (714) were asked to recall the details of the injection provider and venue where they received the last injection (without any time limit). Out of 714 respondents, 458 (64.15%) could remember the last injection received. Approximately 70% (247/354) of the urban area respondents and 59% (211/360) of the rural area respondents could recall the last injection event. Table 4 shows the injection providers and venue of the injection event as recalled by the respondents. Formal sector (doctor, nurse, HA, CMA, AHW etc.) who are trained and have the legal right to provide injections, were most commonly consulted for injections followed by persons working in medical dispensaries. As HA, CMA, and AHW were identified as ‘a doctor’ by many respondents, all of them were included in the formal sector in our study.

**Table 4:** **Injection providers and venue of injection events**

**Characteristics**		**Urban, n = 247 frequency (%)**	**Rural, n = 211 frequency (%)**	**Total (%), n = 458**	*Chi-square (p) value
Injection providers	Formal sector	171 (69.23)	180 (85.31)	351 (76.64)	18.047 (<0.001)
	Medical dispenser	68 (27.53)	30 (14.22)	98 (21.40)	
	Relative	4 (1.62)	0 (0.00)	4 (0.87)	
	Self	4 (1.62)	1 (0.47)	5 (1.09)	
Venue of injection events	Health care facilities	126 (51.02)	143 (67.77)	269 (58.73)	18.698 (0.001)
	Clinic	35 (14.17)	23 (10.90)	58 (12.66)	
	Medical Shop	75 (30.36)	38 (18.01)	113 (24.67)	
	Home	9 (3.64)	2 (0.95)	11 (2.40)	
	School**	2 (0.81)	5 (2.37)	7 (1.53)	

*Chi-square and p value (two-sided) were calculated by Pearson chi-square test comparing urban and rural frequencies; **During national vaccination programs, schools were also used as a vaccination venue.

#### Type of syringe used to administer injections

Most (94.76%) respondents reported that a single use disposable syringe was used for administering injection and the syringe was taken from a sealed packet. A few (4.37%) of the respondents could not recall the type of syringe used. The proportion of respondents reporting the use of sealed-packed, disposable syringes was similar (p = 0.424) in urban and rural areas.

#### Injection administration service charge

Almost half the injection receivers and household heads from urban and rural areas who could remember the last injection events reported that they were not aware of the service charge they paid for injection as the charge was either added along with injection equipment and medicine costs or taken in the form of commodities. The percentage of people unaware about the service charge were higher in urban areas than rural and the difference was statistically significant (p < 0.001). Almost a quarter of the respondents said that they received the injection free of cost while another quarter of respondents could not remember the cost. As the people in rural areas rely more on government health facilities where the services were provided free of cost, fewer people in rural areas reported paying service charge for injection administration compared to urban areas.

#### Incidence of needle stick injury (NSI)

Among the 714 respondents, 19 (2.66%) reported that they had suffered a NSI through an injection needle in the garbage or in the environment. Of these, 13 (68.42%) and 6 (31.58%) were from urban and rural area respectively. Some respondents had sustained the injury while swimming in a river, implying that used syringes might have been disposed of in rivers.

#### People’s perception about injections

When the 714 respondents were asked to name the dosage form they preferred for fever, 564 (79%) said they would prefer oral dosage form like tablet or capsules. In comparison, 5% of respondents preferred injection/intravenous fluid and 16% of respondents shared that they have no particular preference. More rural respondents (7.8% rural Vs 2.3% urban) preferred injections while more urban respondents (90.1% urban Vs 68.1% rural) preferred oral pills for fever and this difference was found to be highly significant statistically (chi square = 63.440, p <0.001).

The reasons given for preferring oral pills over injections were: oral is easy to take (43.1%), an injection is painful/ I am scared of an injection (12.5%), oral pills have less side effects (13.5%), others (15.1%), and do not know (15.8%).

A significant number of people preferred oral pills because of fear of injections.

*“Once we use injection for fever, we become addicted. It will have to be repeated every time we then have fever.”***[Rural Female (RF)-19-5/8]**

*“……. Once we receive injection for fever then pills will not work next time & injection becomes must for future……”***[Urban Female (UF)-25-7/2]**

During FGDs it was found that people had a perception that injection is powerful and should be used in severe (serious) illness only.

*“Injection is for malaria (severe disease) only…. not for general fever.”***[Rural Male (RM)-52-7/5]**

*“Pills are generally prescribed for fever so we take them….. Injection is preferred when there is big fever or patient is admitted in a hospital……”***[Urban Male (UM)-35-1/6]**

*“When we* (the village people) *fall sick…. we use medicinal herbs* (easily available) *or go to traditional healers first and continue our work… Until we become very serious we don’t go to hospitals. When we go to hospital that means, we are very serious and pills will not work…we need some powerful medicines… Yes Injections…”***RM-54-1/8**

Respondents’ perception about the seriousness of disease were duration of illness (time taken to be cured), response to the oral therapy (in serious diseases oral pills are ineffective and injections are used), and actions taken during and as a result of the illness (e.g. admission to the hospital, failing to perform normal duty or taking to bed at home).

Around 6% of the respondents believed that injections are a must for them because they thought that their expectations will be fulfilled only by injections or that they were dependent on injections. Some of the typical expectations or reasons for their preference for injections were as follows:

*“I always suffer from high* (severe type) *fever… hence injection is a must for me….”***RM-59-2/6**

*“Medical* [*dispenser*] *hesitate to give injection in fever. But I need saline* (IV fluid) *for fever…. because it cools [my] body and blood…”***RM-56-3/5**

*“…I am addicted to saline* (IV fluids), *every summer I have to take few bottles [of IV fluids] to feel good for the whole year”***RF-62-2/8**

Few respondents also reported that they prefer injections because they have a perception that injections are longer acting than pills. One female respondent said, *“Swallowing of tablet is difficult and it is bitter too, so I prefer Injection. Injection also acts for longer periods of time… See contraceptive pills have to be taken daily while injectable contraceptive works for three months”***UF-40-2/8***.* In Nepal, injectable contraceptives (depot preparation) which work for three months are a popular choice.

During the survey, 114 (16%) respondents (as mentioned above) said they have no particular preference for a dosage form in fever and would accept whatever their health care professionals prescribed. Some of the common responses were:

*“It is the quality of medicine which counts, not the injection or pill … So anything (pill or injection) is ok, provided they are of quality”***UM-54-3/2**

Despite the reasons reported above, some people reported avoiding injections to prevent complications (risks) associated with injections.

*“Injections transmit diseases…So I avoid injection even a TT* (Tetanus Toxoid injection) *…”***RM-42-4/3**

*“We don’t know what type of syringe is being used…dirty syringe cause adverse effects…… I prefer tablets…”***RM-57-5/4**

*“The injection stored improperly may not work. It requires very good storage condition which tables do not require. Tablets always work…so tablets are better.”***UM-37-4/7**

#### *Knowledge about safe injection practic*e

Most respondents (more than 90%) were aware that diseases may be transmitted when unsterile syringe is used for administering injections or when the same syringe is used to administer injections to more than one person. Table 5 shows that urban respondents were more aware about unsafe injection practice than rural respondents. Some typical responses shared during the FGDs were:

**Table 5:** **Respondents’ knowledge about safe injection practice**

**Issues**		**Urban frequency (%)**	**Rural frequency (%)**	**Total (%)**	*α ^**2**^ **(p) value**
Unsterile syringe can transmit diseases	Yes	345 (97.46)	314 (87.22)	659 (92.30)	26.410 (<0.001)
	No	0 (0.00)	1 (0.28)	1 (0.14)	
	Don’t know	9 (2.54)	45 (12.50)	54 (7.56)	
Sharing syringe can transmit diseases^$^	Yes	341 (96.33)	317 (88.06)	658 (92.16)	16.920 (<0.001)
	No	1 (0.28)	4 (1.11)	5 (0.70)	
	Don’t know	12 (3.39)	39 (10.83)	51 (7.14)	
Sharing syringe between family members also transmit diseases^$^	Yes	326 (95.60)	304 (95.90)	630 (95.74)	0.653 (>0.05)
	No	8 (2.35)	5 (1.58)	13 (1.98)	
	Don’t know	7 (2.05)	8 (2.52)	15 (2.28)	

*Chi-square & p value (two-sided) were calculated by Pearson chi-square test.

*“Injection should be taken from qualified doctor or nurse…… Nowadays a lot of quacks practice* (administer injections)*…… so [it is] difficult to identify who is qualified… One person from our village received injection for one disease and became infected with another disease* (transmitted other disease)*……”***RF-69-3/7**

In some cultures, family members share clothing and take food from the same plate. But they avoid sharing the same with a person outside their family. They may think that sharing the same syringe between family members is acceptable but it should not be shared with people outside the family. To differentiate this, the two statements (marked as $) in Table 5 were asked separately. Table 5 shows that most (92.16%) of the people were aware that syringe should not be shared between two persons and not even between family members.

*“…… previously syringes were reused but nowadays it is said* (HCW and Radio) *that it* (syringe) *should be used only for one person [single person]….”***UF-71-5/8**

“… *the breath of people differs from person to person … Even sharing clothes or jutho* (sharing food from the same plate) *will transfer diseases, then why not syringe. Yeah… of course sharing same injection will transfer diseases……”***UM-69-8/3**

Among the 659 respondents who stated that diseases are transmitted by the use of unsterile syringes, 541 (82.09%) could name one or more diseases transmitted by administering injections using unsterile syringes. The percentage of respondents naming one, two, three, four, and five or more correct diseases were 45.1%, 24.1%, 10%, 2.3%, and 0.61% respectively. Urban respondents who were able to name a greater number of diseases transmitted by the use of unsterile syringes, were significantly higher (p < 0.001) than respondents from rural stratum.

Most respondents (77.69%) named HIV/AIDS as one of the diseases transmitted by the use of unsterile syringes (Table 6).

**Table 6:** **Peoples’ knowledge about diseases that might be transmitted through unsterile syringes**

**Disease transmitted#**	**Urban, n = 345 frequency (%)**	**Rural, n = 314 frequency (%)**	**Total (%) n = 659**	*Chi-square (p) value
HIV/ AIDS	291 (84.35)	221 (70.38)	512 (77.69)	18.500 (<0.001)
Hepatitis/ Jaundice	77 (22.32)	14 (4.46)	91 (13.81)	44.057 (<0.001)
Tetanus	62 (17.97)	25 (7.96)	87 (13.20)	14.372 (<0.001)
Tuberculosis	40 (11.59)	29 (9.24)	69 (10.47)	0.975 (>0.05)
Malaria	14 (4.06)	12 (3.82)	26 (3.95)	0.024 (>0.05)
Hepatitis B	10 (2.90)	1 (0.32)	11 (1.67)	6.667 (<0.05)
Hepatitis C	3 (0.87)	0 (0.00)	3 (0.46)	2.743 (>0.05)
Other diseases	53 (15.36)	13 (4.14)	66 (21.02)	22.972 (<0.001)

#The question was multiple response so the total is more than 100%; *Chi-square and p value (two-sided) were calculated by Pearson chi-square test comparing urban and rural frequencies.

#### Access to mass media and their use for health information

Radio, Television, Internet, and Newspaper were accessible to 57%, 81.5%, 21.0%, and 44.1% of respondents, respectively. People in urban areas had significantly more mass media accessible to them compared with those in rural areas.

Mass media are an important source of health related information. Out of the 714, 553 (77.45%) respondents reported that they depend on at least one mass medium to get health related information. Tendency to use media for health related information was more in urban population compared to rural population. Approximately 91% (322/345) and 64% (231/360) of respondents from urban and rural areas respectively, depended on at least one mass media for health related information. Most respondents (almost 66%) from urban area were using two or more media sources for health related information while 48% of rural respondents were fortunate to have access to the same (p < 0.001).

Television was an important mass medium used for health related information by the respondents and 77% of respondents (83% urban Vs 69% rural, p < 0.001) used television as a source of health information.

## Discussion

In the present study in at least one out of three households, one or more members received at least one injection during the last three months and 10% of the sampled population received injection/s during the last three months. The injection use was more in rural population compared to urban.

### Injection use

The overall frequency of injections in our study was 2.37 injections per person per annum. This is more than that reported in African (2.0 – 2.2), American (1.7 – 1.9) and South East Asian B (2.1) regions [2] but is less than that reported in an Indian study (2.9) [35], and South East Asia Region D (4.0), including Nepal [2]. The injection frequency is half of that observed in Cambodia (5.9) [36].

A high proportion (97.13%) of injections used in Kaski district were given for therapeutic reasons which is similar to the global data, (95%) [3] but greater than the data from India (82.7%) [35]. Health care facilities visited for basic health care needs by the people in urban and rural areas were significantly different. Government primary health care facilities or clinics of the HCWs working at those facilities were mostly preferred by rural people while medical dispensary was preferred by urban people. The medical dispensaries (community pharmacies) were preferred by the community because a variety of medicines [20] and services [21] were available at the dispensaries, and people found them to be cheaper [20,37]. As both health care facilities (government health care facilities and medical dispensaries) were rarely supported by laboratory investigations, in most cases the diagnosis and treatment was based on only clinical presentation which may not be an ideal situation. This highlights a need for a Standard Treatment Guideline/Strategies for common illness in primary health care facilities, however, the Standard Treatment Strategies in Nepal has not been revised since 1999 [38].

### Injection providers and venue

People reported that both the formal sector [including doctor, nurse and other paramedicals (HA, CMA, ANM etc.)] and informal sector (e.g. Ayurvedic HCWs, Medical dispensers and pharmacists) were consulted for medical problems and they received injections from the providers as well. As injection use provides financial rewards and status to providers [7,29], the informal sectors may have been attracted towards injection use because they have less status and money compared to physicians. The informal sector is neither qualified nor formally trained for injection administration hence the injection provided by them may be neither safe nor rational [29].

Implementation of interventions for safe injection in the formal health care system is easier because it is under the direct control of the government. The challenges increase if injections are provided outside the formal health care system [1]. As more people in urban areas in our study received injections from medical dispensaries (outside the formal health care system) than the rural population, interventions promoting safe injection practice might be more challenging in urban than in rural areas.

Almost a quarter of respondents in our study received their injections at medical dispensaries which was similar to the results of a study from Pokhara city [21]. The study done by Gyawali et al. in 2012 at community pharmacies of Pokhara city showed that the pharmacies (dispensaries) were not well equipped and most working manpower of the pharmacies were neither qualified nor trained for injection practices [21]. Furthermore, in our study we found that medical dispensers were considered as doctors and people preferred consulting them for treatment of minor illness. The injections administered by these unqualified personnel might be unsafe and non-rational [29] but these personnel could be influenced by the government for safe injection practice.

A qualitative study conducted in central Nepal [23] reported that grocery shops were also used as the venue for injections but in our study this was not reported. Similarly, a wide variety of venues including public schools, teashops, and temples were reported to be used for immunization in Nepal [12]. As schools are also used as immunization venue for national immunization program in Nepal [19], the schools were reported as a venue in our study as well.

### Needle Stick injury (NSI) caused by improperly disposed of syringes

Safe disposal of used injection equipment is vital to promote safe injection practice. Unsafe disposal of sharps (used injection equipment) exposes the community to needle stick injury (NSIs) [39]. A few respondents (2.66%) in our study reported that they were injured by an injection needle in the garbage or in the environment. This proportion is more than that reported in a study based in Pakistan (0.2%) [31].

### Knowledge and perception about injection practice

It was found that the knowledge about injection practice was better among urban dwellers than rural ones. Consistent with the finding from a previous study among health policymakers [25], almost all injection receivers (94.76%) who recalled the last injection event reported that plastic syringes taken from a sealed packet were used for providing the injection. In Pakistan, 73% of patients did not know whether the needle and syringe used to inject them were sterile [40]. This may indicate that the people of Kaski were aware and consciously checked the quality of the syringe (new syringe taken from sealed pack) used. Empowering the community to question the need for an injection and whether the syringe being used was new, can have a meaningful impact in improving injection safety [41], as using sterile syringe for administering rational injection is important for the safety of injection recipients [10,15]. Unfortunately, the quality of the injection equipment available in Nepal is not monitored [25] so the quality of the equipment may not be assured.

In our study, fever was used as the condition to study the preference for dosage form (oral or injection) because the illness could be described accurately using local terms and it is also an illnesses which is either self-limiting or could be treated by oral therapy. The respondents shared that they preferred oral pills because the pills were easy to administer, not painful and safe (no additional side effects like injection). The respondents in our study do not prefer injections because they had a perception that injection produces dependence i.e. if an injection is used for an illness, the next time when a person gets similar illness oral pills may not work and injection becomes necessary.

Most respondents in our study had a perception that injections are powerful and should be used only in severe cases (major illness), in weak patients, and/or when oral pill is not effective. They also had a perception that fever is generally a minor illness so pills are sufficient and injections are not required. The incidences of diseases such as encephalitis, malaria, kala-azar and dengue, which are fatal and produce fever as a symptom are either low or absent in Kaski district [19]. This may be one reason why fever was considered as a minor illness. However, the incidence of such diseases is more frequent in the Terai region [19] so the perception of fever among people from that region might be different. Hence, the results should be extrapolated with caution to the Terai region of the country.

A study from Pakistan showed that majority of community participants [82% (n = 249)] preferred injections [41]. But in our study, only 5% preferred injection. The proportion of people preferring injection in our study was almost the same as the global data (5%) [29] and less compared to a study from Pakistan [40] where 44% of patients preferred injectable medicine. Global data show that injections are preferred because they are thought to be powerful, fast acting, latest technology and other reasons [7,29,35]. The few respondents in our study who preferred injection did so because they thought injections are longer acting than oral pills or they are dependent on the injection. This might because of their perception about injectable contraceptives (depot preparation) which work for three months and also due to the placebo feeling of wellbeing for few months after receiving IVF. Few of the respondents (16%) also reported that they would like to leave decisions about the dosage form to HCWs which was similar to a recent Pakistani study where almost 18% of patients left the decision to prescribers [41].

More respondents in rural areas preferred injection compared to urban (7.8% rural Vs 2.3% urban). This might have influenced use of injections; hence number of people receiving injection and use of injection were higher in rural compared to urban respondents.

More than three quarters of the respondents were aware and could name at least one correct disease that may be transmitted when unsterile (dirty) syringe is used for administering injections or when a syringe is shared to administer medicine to more than one person. Higher number of respondents from urban area (90.16%) than rural respondents (73.25%) could name one or more diseases transmitted by such practice. This might be due to the communication of the risk of HIV infections associated with injection through HIV/AIDS prevention and care programs as mentioned by the health policymakers of Nepal [25]. Some of the respondents had a perception that sharing the same syringe between two or more persons other than family members may transmit diseases but not if shared among family members. They were of the opinion that all family members had a similar structure, physiology and even diseases. This perception was similar among both urban and rural dwellers.

Most respondents (82%) had access to television followed by radio and newspaper and these media in the same sequence were used as sources for health related information. Similar response was also reported in Pakistan where 79% of respondents reported that television was the important community source for health information [41]. Television and radio could be used more extensively to provide information (educating population) about safe injection practice to people of remote areas.

Significantly higher literacy rate, accessibility to multiple mass media and tendency to use the media as a source of health related information in urban respondents compared to rural ones might have increased awareness and knowledge about safe injection practice among urban respondents. Good awareness about the association between injections and infections among the general population may lead to demand for safe injections by the community. The awareness also leads to a demand for use of sterile injection equipment (syringes) for injections. As the people were aware of single use of syringe and had some knowledge about the diseases transmitted through unsterile syringes, it could be postulated that the syringe or needles were not reused in the absence of sterilization in Kaski which ultimately may lead to safe injection practices. However, receiving injection from unqualified personnel at venues lacking facility for the injection could lead to unsafe injection practice.

### Strengths of the study

Injection use studies conducted among patients visiting or admitted in health care facilities may report the injections administered by formal providers (mostly qualified and trained) only. The present study has quantified the injection use in the community and could be considered as reliable data because it included all injections administered at all types of health care facilities (primary, secondary, tertiary etc.), at home and in other places by all types of providers (formal, informal, quacks and self). This study has identified different types of injection providers (qualified/trained, unqualified/untrained and quacks) prescribing and administering injections to the community. The results of this study may serve as the baseline for conducting studies in the community regarding injection usage in other districts and the western development region of Nepal.

### Limitations of the study

The study was carried out in one of the hill districts of the country so the results may not represent other non-hill regions because the disease patterns, peoples’ perception towards common diseases & injection efficacy, access to health care facilities, burden of illness etc. may differ. As the FGD participants were not selected randomly and very few females participated, the qualitative data from FGDs may not be representative if considered alone. Hence, the qualitative results should be considered along with the quantitative results. Checking of the transcript by the FGD participants could not be done because of time constraints. All the FGDs were not recorded. As with all survey questionnaires, participant recall bias may have occurred.

## Conclusion

Use of disposable equipment, less preference for injections by the people, and high awareness about the association between injections and injection borne infections among the study population is encouraging with regard to safe injection practice. In urban areas, there was a greater awareness of safe injection practice and the use of and preference for injections was lesser compared to rural areas. Unfortunately, people were not aware of the importance of qualified injection providers for safe injections and were receiving injections from unqualified personnel. The number of people receiving injection from informal providers was more in urban compared to rural area.

### Recommendations

As unsafe injection practices result in a substantial burden of preventable blood-borne diseases, assuring safe injection practice in developing countries is important. Empowering the community to enquire about the rationality of an injection and demand for safe injections could be vital in producing improvement in the situation. Awareness about the importance of obtaining injections only from qualified providers should be increased. Similarly, monitoring the quality of the injection equipment available in Nepal is also needed. Interventions to improve injection practice may be more challenging in urban compared to rural areas because more urban residents receive injections from the informal sector. Further research to identify the injection types and its quantity administered by informal sectors is also required to increase the effectiveness of future interventions.

## Additional file

Additional file 1:
**Basic Household Information Form.**

## Abbreviations

AHW: Auxiliary Health Workers
ANM: Auxiliary Nursing Midwifes
CHW: Community Health Workers
CMA: Community Medical Auxiliary
DoHS: Department of Health Services
EA: Enumeration Area
FGD: Focus Group Discussion
HA: Health Assistant
HBV: Hepatitis B Virus
HCV: Hepatitis C Virus
HCW: Health Care Worker
HH: Household Head
HIV: Human Immunodeficiency Virus
HP: Health Post
NSI: Needle Stick Injury
PHCC: Primary Health Care Centers
RF: Rural Female
RM: Rural Male
SHP: Sub Health Post
UF: Urban Female
UM: Urban Male
VDC: Village Development Committee
WHO: World Health Organization
