# Supplementary material for: Injection practice in Kaski district, Western Nepal: a community perspective
Source: BMC Public Health. 2015 Apr 29;15:435. doi: 10.1186/s12889-015-1775-5 (PMC4425888; doi:10.1186/s12889-015-1775-5)
Supplement: Additional file 1: — Basic Household Information Form. [file 12889_2015_1775_MOESM1_ESM.doc]

| S. No. | Age | Gender | Education | Religion | Ethnicity | Occupation | Household Head | Injection receiver |
| --- | --- | --- | --- | --- | --- | --- | --- | --- |
| 1. |  |  |  |  |  |  |  |  |
| 2. |  |  |  |  |  |  |  |  |
| 3. |  |  |  |  |  |  |  |  |
| 4. |  |  |  |  |  |  |  |  |
| 5. |  |  |  |  |  |  |  |  |
| 6. |  |  |  |  |  |  |  |  |
| 7. |  |  |  |  |  |  |  |  |
| 8. |  |  |  |  |  |  |  |  |
| 9. |  |  |  |  |  |  |  |  |
| 10. |  |  |  |  |  |  |  |  |
| 11. |  |  |  |  |  |  |  |  |

**BASIC HOUSEHOLD INFORMATION FORM**

**Location:**  **Code No:**

**…………………………**

**Name of interviewer: Signature of interviewer**
